# Scaling theory of electric-field-assisted tunnelling

**DOI:** 10.1098/rspa.2014.0014

**Published:** 2014-07-08

**Authors:** Thomas C. T. Michaels, H. Cabrera, D. A. Zanin, L. De Pietro, U. Ramsperger, A. Vindigni, D. Pescia

**Affiliations:** 1 Department of Chemistry, University of Cambridge, Lensfield Road, Cambridge CB2 1EW, UK; 2 Laboratory for Solid State Physics, ETH Zurich, Zurich 8093, Switzerland

**Keywords:** scaling, Fowler–Nordheim tunnelling, electron microscopy

## Abstract

Recent experiments report the current (*I*) versus voltage (*V*) characteristics of a tunnel junction consisting of a metallic tip placed at a distance *d* from a planar electrode, *d* varying over six orders of magnitude, from few nanometres to few millimetres. In the ‘electric-field-assisted’ (or ‘field emission’) regime, as opposed to the direct tunnelling regime used in conventional scanning tunnelling microscopy, all *I*–*V* curves are found to collapse onto one single graph when *d* is suitably rescaled, suggesting that the current *I*=*I*(*V*,*d*) is in reality a generalized homogeneous function of one single variable, i.e. $I=I(V\cdot d^{-\lambda })$, where λ being some characteristic exponent and I(x) being a scaling function. In this paper, we provide a comprehensive explanation—based on analytical arguments, numerical simulations and further experimental results—for the scaling behaviour that we show to emerge for a variety of tip–plane geometries and thus seems to be a general feature of electric-field-assisted tunnelling.

## 1. Introduction

**Figure 1.**
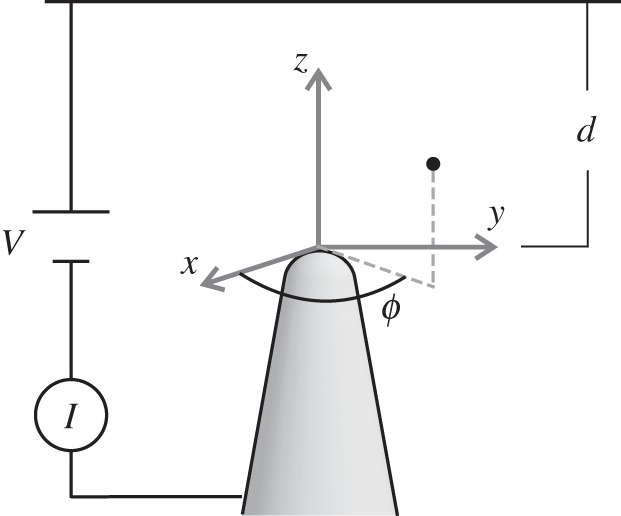
Schematic of the tunnel junction. The emitter (shaded in grey) possesses rotational symmetry with respect to the *z*-axis and carries the *x*,*y*,*z*-coordinate system at its apex. The azimuthal angle *ϕ* used throughout the paper is defined as the angle formed by the *x*-axis and the projection onto the *xy*-plane of the coordinate vector of an observation point in space (black dot). In a typical *I*–*V* experiment, a positive voltage *V* is applied to the planar counterelectrode residing at a distance *d* from the emitter and the resulting current *I* flowing from the planar counterelectrode to the emitter (meaning that the electrons flow from the emitter to the planar counterelectrode) is recorded.

A sharp tip approached vertically to a conducting surface at subnanometre distances and biased with a small voltage with respect to the surface builds a junction across which electrons can be transferred from the tip apex to the nearest surface atom (or vice versa) by direct quantum-mechanical tunnelling. Such a junction is used, e.g., in scanning tunnelling microscopy (STM) for imaging the surface topography with the spectacular atomic spatial resolution that was awarded the Nobel Prize in 1986 [1–3]. When the distance *d* between tip and collector is increased, one enters the electric-field-assisted tunnelling regime [1–4], where the current is dominated by electrons emitted from the (typically sharp) tip into the vacuum region residing between the tip apex and the target through a classically forbidden zone enveloping the tip apex (figure 1). Such a regime is, for instance, the one underlying the topografiner technology [5,6]—an imaging technique which was the precursor of STM but was abandoned, probably because of the enormous success of STM. Electric-field-assisted quantum tunnelling is also widely used in recent and less recent developments in micro- and nano-electronics [7,8]. Recent experiments [9] in the regime of electric-field-assisted tunnelling suggest a remarkable scaling invariance of the current flow with respect to changes in the tip-to-collector distance *d* by several orders of magnitude (from a few nanometres to a few millimetres). This scaling invariance was detected by observing the collapsing of the family of *I*–*V* curves, measured at various distances *d*, onto one single curve when the voltage was suitably rescaled with a scaling factor *R* depending on *d* through a power law *R*∼*d*^−λ^, i.e. $I=I(V\cdot d^{-\lambda })$, I(x) being a *scaling function*. Such a scaling invariance—well known, e.g., in the field of critical phenomena [10]—is certainly not realized in the direct tunnelling regime [1], and it is also not usual in solid-state electronics, so that its observation is yet somewhat surprising and unexplained. It is the scope of this paper to provide a comprehensive explanation of the experimentally reported [9] scaling invariance by using analytical and numerical arguments, and by introducing further experimental results that establish the scaling behaviour as a systematic property of junctions in the electric-field-assisted tunnelling regime. The paper is organized as follows.

In §2, we consider the electrostatic problem of a sharp metallic tip, forming one side of the junction, which is approached vertically at a distance *d* by a planar counterelectrode, forming the other side of the junction. We formally solve the associated Laplace equation and find analytical expressions for the electrostatic potential *Φ*(*x*,*y*,*z*) by considering tip and plane as equipotential boundaries, with the tip being at ground and the plane being at a positive potential +*V* . Within our treatment, we consider only highly symmetric, realistic tip shapes. The wording ‘highly symmetric realistic’ describes here the fact that the geometries considered in §2 are (i) close to the shape one expects for ‘real’ tips (as revealed by a systematic tip imaging via light and electron microscopy [9]) and (ii) sufficiently symmetric so that the electrostatic problem can be solved to a large extent analytically. On the one hand, this choice helps the reader to follow the main arguments without relying on numerical results only, and on the other hand it is well-known that unequivocally revealing power laws in experiments and numerical computations is a very difficult task, but it is quite straightforward within an analytical framework. The main result of §2 is the *conjecture* that in the vicinity of the tip end the ‘almost’ singularity associated to the tip apex introduces a power-law dependence of the potential on *d* and *V* of the form:

1.1 $$\Phi (x,y,z;d,V)∼V\cdot d^{-\lambda }\cdot \tilde{\Phi }(x,y,z),$$

where λ is a characteristic exponent that depends on the geometrical details of the tip and on the range of distances *d* (see the detailed discussion in §2). $\tilde{\Phi }(x,y,z)$ is some function containing only the coordinates (*x*,*y*,*z*), (*x*,*y*,*z*) indicating points residing very close to the tip apex so that *z*≪*d*. The meaning of the symbol ‘∼’ in equation (1.1) and other equations in this paper will be discussed in detail in §2. Note that, for example, for a ‘planar’ tip (in which case we should no longer speak of ‘sharp tip’, of course) λ assumes the trivial value of 1.

In §3, we use the one-dimensional Jeffreys–Wentzel–Kramers–Brillouin approximation of quantum tunnelling [11] to compute the tunnelling current density *J* from the tip into the planar electrode in the presence of a potential that behaves as given in equation (1.1). The main result of this section is to show that, for an emitter subject to the electrostatic potential worked out for different geometries in §2, the scaling behaviour of the potential implies that the current density *J* becomes a function of one single variable

1.2 $$J(V,d)=J(V\cdot d^{-\lambda }),$$

where λ is the same exponent appearing in equation (1.1). We obtain an analytical expression for the scaling function J(x) both for λ=1 [12–15] and for λ≠1. These exact scaling results led us to identify a fundamental length scale *Λ*_*φ*_—the De Broglie wavelength associated with the maximum height of the tunnelling barrier (*φ*)—and an effective barrier width Δ that determine the leading behaviour of the current density

1.3 $$J(\Lambda_{\varphi },\Delta )∼e^{-\Delta /\Lambda_{\varphi }}.$$

This equation is certainly true for the few, highly symmetric models of electric-field-assisted tunnelling discussed in this paper, but it might hold approximately in general and for both direct and electric-field-assisted tunnelling.

In §4, we present experimental data and numerical results from simulations that corroborate the scaling hypothesis and establish its systematicness. Moreover, we report on the experimental observation of small deviations from the scaling hypothesis and discuss their possible origin. Finally, we *attempt* to combine experiments, numerical results and scaling hypothesis into a proposal for a functional dependence J(x) that explains most of the data.

The appendices A and B present the mathematical details of the calculations leading to the results summarized in the main text. The scope of these appendices is to allow the verification of our computations which, reporting about the relatively recent idea of scaling in the tunnelling regime (Cabrera *et al*. [9]), might evoke some (salutary) scepticism.

## 2. Electrostatics of the junction in the electric-field-assisted tunnelling regime

Within the purpose of this paper, we consider a conducting tip as an infinitely long object with ‘small’ cross section and ending with a more or less sharp apex. The tip is kept at 0 potential and it is placed vertically at a distance *d* from a conducting plane held at potential +*V* (figure 1). The aim of this section is to evidence the scaling behaviour of the potential *Φ*(*x*,*y*,*z*) on the parameters *V* and *d*, which are typically imposed experimentally [9]. If *Ω* denotes the region of space excluding the tip and the plane, then the electrostatic problem defining the *electrostatic* potential *Φ*(*x*,*y*,*z*) is a well-defined Dirichlet problem and reads

2.1 $$\begin{array}{lll} & \nabla^{2}\Phi =0 & \text{in }\Omega \\ & \Phi =0 & \text{on the surface of the tip}\\ & \Phi =+\Phi_{d} & \text{on the plane }\\ \text{and}\; & |\Phi (x,y,z)|\leq +\Phi_{d} & \forall (x,y,z)\in \Omega , \end{array}\}$$

where the last equation follows from the maximum principle of harmonic functions. The solution of equation (2.1) is unique and can be computed, at least numerically, in the entire space *Ω*. However, for the purposes of §§3 and 4, where analytical expressions for the tunnelling current density will be derived, only the behaviour of the potential in the very vicinity of the tip apex and along the tip axis is required [1,11]. In this section, we therefore focus only on the behaviour of *Φ* (*x*=0, *y*=0,*z*) for small *z*. In the following, we summarize the main results. The details of the calculations are presented in appendix A.

**Remark**

The boundary condition on the tip assigns a uniform value of the electrostatic potential *Φ*_*d*_ to the tip surface. We use the symbol *V* (figure 1) to denote the experimental voltage recorded during the measurements of the experimental *I*–*V* characteristics, instead. We acknowledge that *V* contains an electrostatic contribution (which can be considered to be the *Φ*_*d*_ defined here), but also a contribution due to work function difference between tip and planar electrode, not considered in these sections. Moreover, real emitters might have a non-uniform spatial distribution of work functions, which is also neglected in this section. We point out that the difference between *Φ*_*d*_ and *V* is typically 1 V or less, so that the distinction between them can usually be neglected. However, for small *d* and for small *V* , this difference might be a source of small deviations from practical scaling (experimental deviations are indeed observed and reported later).

### (a) Conical and cuspidal tips

**Figure 2.**
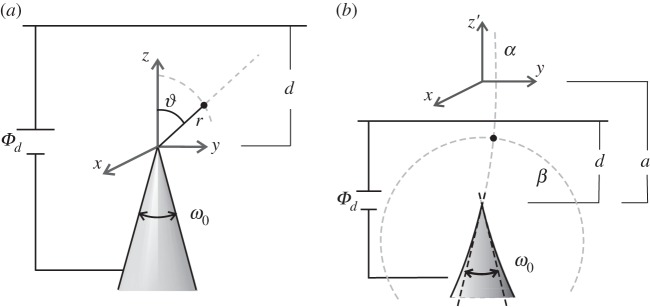
(*a*) Emitter with conical profile. The coordinates of a point (black dot) in the *y*−*z*-plane are described by the intersection of a line of constant spherical coordinate *ϑ* and a circle of radius *r* centred at the origin. The full angle of aperture of the cone *ω*_0_ is indicated in the figure. (*b*) Emitter with cuspidal profile. The coordinates of a point (black dot) in the *y*−*z*-plane are described by the intersection of a circle of constant bispherical coordinate *β* (circle centred at the focal point *z*′=*a*) and a circle of constant bispherical coordinate *α* (a circle with centre along the axis *z*′=0). Tangent lines to the cusp at the tip apex define an angle *ω*_0_, which can be identified as the full angle of aperture of the cusp.

These geometries describe the shape of real tips on the micrometre scale [9], but contain a true singularity at the tip apex (figure 2). For both distant (*d*≫0) and near (*d* small) planes, we have proved in ref. [9] (see also appendix A for more details) that the potential in the vicinity of the tip apex has a leading term of the form

2.2 $$\Phi (z,d)∼\Phi_{d}\cdot {\left(\frac{z}{d}\right)}^{\lambda_{1}},$$

where the exponent λ_1_ is defined as the smallest index λ for which the Legendre function *P*_λ_(*x*) has a zero at $x=\cos(\pi -\omega_{0}/2)$. For small angles of aperture, the exponent λ_1_ is given approximately by [16,17] $\lambda_{1}(\omega_{0})≅{\left[2\ln(2/\omega_{0})\right]}^{-1}$, whereas for *ω*_0_=*π*, corresponding to a planar emitter, we have λ_1_=1 [16,17].

**Remark**

Quoting from ref. [18], pp. 39–40, almost ‘verbatim’ but ‘mutatis mutandis’, we point out that the relation *Φ*(*z*,*d*)∼*Φ*_*d*_⋅(*z*/*d*)^λ_1_^ does not imply the relation *Φ*(*z*,*d*)=*A*⋅(*z*/*d*)^λ_1_^. In general, we find (see ref. [9] and appendix A) that there is an infinite number of additional correction terms of the type (*z*/*d*)^λ_*k*_^, with—and this is the crucial point—λ_*k*_>λ_1_. In the limit (z/d)→0—which is the asymptotic case considered for discussing ‘scaling’—these additional terms become infinitesimally small with respect to the leading one (*z*/*d*)^λ_1_^, so that we can write

2.3 $$\lim_{z/d\to 0}\frac{\Phi (z,d)}{\Phi_{d}\cdot {\left(z/d\right)}^{\lambda_{1}}}=\mathrm{const}.,$$

which is equivalent to writing *Φ*(*z*,*d*)∼*Φ*_*d*_⋅(*z*/*d*)^λ_1_^. Equation (2.3) establishes the significance of the symbol ‘∼’ and our use of it in relation to the concept of ‘scaling’.

### (b) Sphere-on-the-cone and hyperboloid of revolution

**Figure 3.**
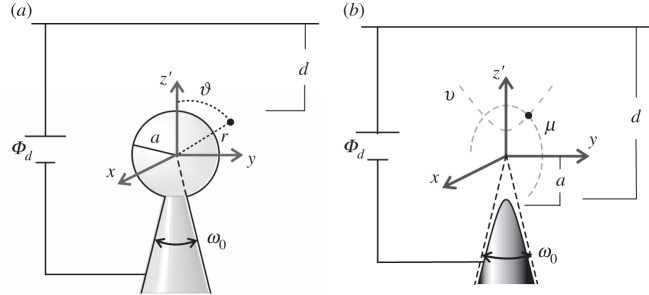
(*a*) Small sphere of radius *a* terminating a conical profile with aperture angle *ω*_0_. (*b*) Hyperboloid of revolution. A point (black dot) in the *z*′−*y*-plane is the intersection of a line of constant prolate spheroidal coordinate *v* (an ellipse with focal points at ±*a* along the axis *z*′) and a line of constant spheroidal coordinate *ν* (a hyperbola with focus at *z*′=*a* or *z*′=−*a*). The asymptotes of the hyperboloidal profile define the full angle of aperture of the tip *ω*_0_.

The sphere-on-the-cone model is particularly suitable for mimicking a rounded tip with overall conical shape (figure 3). In fact, the sphere-on-the-cone model terminates the cone with a small sphere of radius *a*. Hyperboloids of revolution are suitable to mimic rounded tips with asymptotic conical shape. They have, in fact, two asymptotes that can be used to define a full angle of aperture *ω*_0_. The two asymptotes meet at a point in front of the apex that is the intersection between the *z*′-axis and the so-called confocal plane. The focal length *a* is the distance between this point and the focal point of the hyperboloid, which is located within the tip on the tip axis. In contrast with conical and cuspidal tips, the scaling properties of the leading potential term close to the tip apex for the sphere-on-the-cone and the hyperboloid of revolution depend on whether the plane is ‘distant’ (*d*≫*a*) or ‘near’ (*d*≪*a*). In particular, we obtain

2.4 $$\begin{array}{lll} & \Phi (z,d)∼\Phi_{d}\cdot \frac{z}{d} & \text{for}\;d\ll a\\ \text{and}\; & \Phi (z,d)∼\Phi_{d}\cdot {\left(\frac{a}{d}\right)}^{\lambda_{1}}\cdot \frac{z}{a} & \text{for}\;d\gg a, \end{array}\}$$

where λ_1_ is the same exponent found for conical and cuspidal tips.

### (c) Paraboloid of revolution

**Figure 4.**
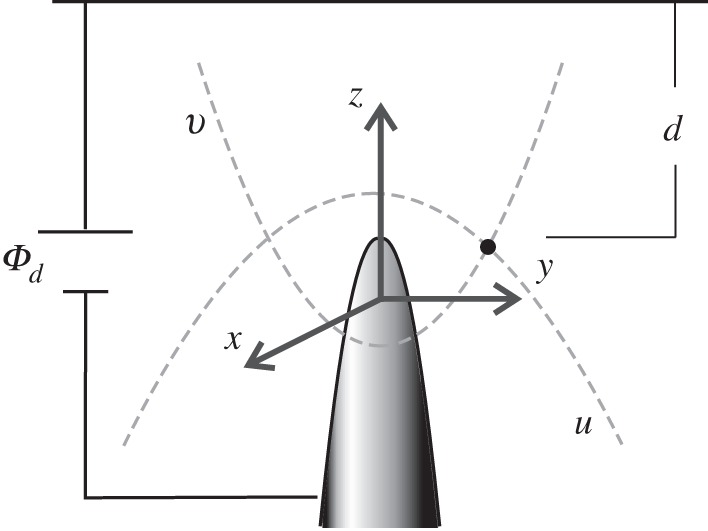
Paraboloidal emitter. A point (black dot) in the *z*−*y*-plane is the intersection of a line of constant paraboloidal coordinate *u* (a downward oriented parabola) and a line of constant paraboloidal coordinate *v* (an upward oriented parabola). The tip apex is at a distance *R*_0_/2 from the origin of the coordinate system, where *R*_0_ is the radius of curvature of the tip.

A paraboloid of revolution is characterized by a radius of curvature *R*_0_, but it does not have a characteristic angle of aperture and therefore misses one essential characteristic of the tips used, for example in ref. [9] (figure 4). However, it provides an interesting limiting case

2.5 $$\begin{array}{lll} & \Phi (z,d)∼\Phi_{d}\cdot \frac{z}{d} & \text{for}\;d\ll R_{0}\\ \text{and}\; & \Phi (z,d)∼\Phi_{d}\cdot \frac{2}{\ln(2d/R_{0})}\cdot \frac{z}{R_{0}} & \text{for}\;d\gg R_{0}. \end{array}\}$$

In fact, by exploiting the identity $\lim_{\mu \to 0}((x^{\mu }-1)/\mu )=\ln x$, we might consider the logarithmic dependence on *d* in equation (2.5) as a special case of equation (1.1) when λ→0.

### (d) General tip geometry

Equations (2.2), (2.4) and (2.5) provide analytical expressions for the potential along the tip axis, ready to be used for computing the tunnelling current (§3). However, the scaling behaviour with *Φ*_*d*_ and *d* observed along the tip axis can be extended by continuity to a small neighbourhood of the tip apex as well, yielding equation (1.1). Moreover, the scaling hypothesis, equation (1.1), has been verified explicitly for a restricted number of highly symmetric geometries, but we *propose* that it might have general validity.

## 3. The tunnelling current density in the presence of a non-trivial exponent λ_1_

**Figure 5.**
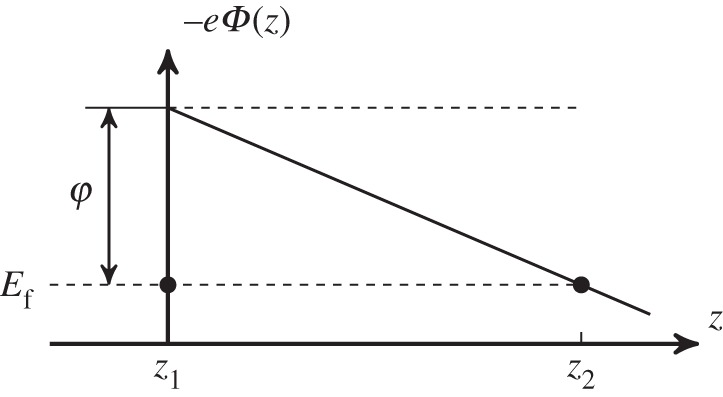
Sketch of the one-dimensional tunnelling barrier profile along the tip axis, showing the potential energy originating from the electrostatic potential for the case of a linear dependence on *z*, for simplicity. This sketch is used to define some quantities used in the bulk of the section. *z*_1_ and *z*_2_ are the points at which the potential energy −*eΦ*(*z*) crosses the Fermi level, with |*z*_2_−*z*_1_| being the width of the potential barrier. *φ* is the work function of the tip.

One of the most remarkable results of §2 is that, even if the planar electrode is a ‘distant’ one, the boundary condition on the plane determines the electrostatic potential in the vicinity of the tip apex, where electric-field-assisted (or field emission) quantum tunnelling occurs. Having shown some scaling properties of the electrostatic potential with *d*, now we would like to address the question whether these scaling properties affect the field emission process at all. In this section, we compute the tunnelling current density for a very simplified model of electric-field-assisted quantum tunnelling, which only involves the presence of the pure electrostatic potential within the tunnelling barrier (figure 5). We also assume, for the sake of simplicity, the purely one-dimensional model used, for example, in ref. [1], which foresees free electrons within the tip, and is therefore strictly applicable only to a large flat planar emitter [19]. For very small radii of curvature—in particular for the conical and cuspidal models—quantum confinement can, for example, occur and the electron energies might become quantized [20]. We also neglect the image potential correction to the electrostatic potential energy, which is known to substantially lower the barrier height and to modify by orders of magnitude the current density [1,11]. Thus, owing to these assumptions, the calculations performed in this section are far from being realistic. Nevertheless, we would like to point out that the scaling properties derived in this section on the base of the pure electrostatic potential are actually obeyed by the experimental data presented in ref. [9] and in this paper (with the limitations discussed in §3*a*). Given this agreement with experiments, we suspect (but cannot prove it!) that all the elements neglected by our simple—perhaps trivial, model—are not really modifying the scaling properties obtained from the pure electrostatic potential. Of course, we are convinced that this last sentence will raise some controversial discussion, but we feel that it might justify the publication of the results presented in this section.

Within our simple, not-so-realistic-model, the current density along the tip axis can be written as [1]

3.1 $$|J_{z}|=e\int_{0}^{\infty }dv_{z}\cdot v_{z}\cdot \rho (v_{z})\cdot D(v_{z}),$$

where −*e* is the charge of the electron, *v*_*z*_ is the *z*-component of the velocity of the free electrons within the metal and *dv*_*z*_*ρ*(*v*_*z*_) is the number of electrons per unit volume with velocity between *v*_*z*_ and *v*_*z*_+*dv*_*z*_. Finally, *D*(*v*_*z*_) is the transmission coefficient of tunnelling describing the probability that an electron with velocity *v*_*z*_ overcomes the potential barrier. By inserting the suitable (free electron like) expression for *ρ*(*v*_*z*_) [1,11] in equation (3.1) and by using the standard Jeffreys–Wentzel–Kramers–Brillouin approximation of quantum tunnelling [1,11], one obtains the following result:

3.2 $$\begin{array}{rl} & |J_{z}|=J_{0}(E_{f})\cdot e^{-G(E_{f})}\\ & G(E_{f})=\left(2\sqrt{\frac{8\pi^{2}m}{h^{2}}}\int_{z_{1}}^{z_{2}}\sqrt{-e\Phi (z)-E_{f}}\;dz\right)\\ \text{and}\; & J_{0}(E_{f})=\frac{4\pi em}{h^{3}}\cdot {\left(\sqrt{\frac{8\pi^{2}m}{h^{2}}}\int_{z_{1}}^{z_{2}}\frac{1}{\sqrt{-e\Phi (z)-E_{f}}}dz\right)}^{-2}, \end{array}\}$$

where *E*_f_ is the Fermi energy of the tip, −*eΦ*(*z*) is the potential energy of the electron within the tunnelling barrier, *m* is the mass of the electron and *G*(*E*_f_) is the so-called Gamov exponent. The integration limits *z*_1_ and *z*_2_ are the zeros of the quantity −*eΦ*(*z*)−*E*_f_ (figure 5).

We now summarize the main scaling results obtained for the current density by assuming a potential that satisfies the scaling hypothesis derived in the previous section. The details of the derivation are described in appendix B.

### (a) Non-analytic potential: conical and cuspidal tip

These two geometries for the tip provide a special case. First, the apex represents a geometrical singularity where it is difficult to imagine that a ‘current density’ is well defined, as the area of the apex itself is zero. Second, the electrostatic potential is non-analytic within the tunnelling barrier: *Φ*(*z*)∼*Φ*_*d*_⋅(*z*/*d*)^λ_1_^. The electric field at the apex, e.g., diverges to infinity, so that standard formulae of field emission [1,11,14,15] are useless. We are therefore in the presence of a mathematically difficult problem: ‘a zero area’ of emission and an ‘infinite’ electric field. It is therefore most remarkable that application of equation (3.2) to the non-analytic electrostatic potential (note that the integrals in equation (3.2) can be computed exactly in terms of known mathematical functions (see equations (B3) and (B4))) produces a *finite* current density *J*(*Φ*_*d*_,*d*) which has a remarkable scaling property associated with the scaling behaviour of the electrostatic potential: although there are two independent experimental variables *Φ*_*d*_ and *d*, the current density is a function of one single variable *Φ*_*d*_⋅*d*^−λ_1_^, namely

3.3 $$\begin{array}{rl} |J^{\mathrm{cone}}(\Phi_{d},d)| & =J^{\mathrm{cone}}\left(\frac{e\Phi_{d}}{\varphi }\cdot {\left(\frac{d}{\Lambda_{\varphi }}\right)}^{-\lambda_{1}}\right)\\ \text{and}\;J^{\mathrm{cone}}(x) & =a_{1}\cdot x^{2/\lambda_{1}}\cdot e^{-a_{2}\cdot x^{-1/\lambda_{1}}}, \end{array}\}$$

where *a*_1_,*a*_2_ are dimensionless numbers, containing, for example, natural constants and $J^{\mathrm{cone}}(x)$ is the scaling function. The *Φ*_*d*_⋅*d*^−λ_1_^ scaling agrees with the experimentally observed scaling behaviour [9]. The scaling function will be discussed in §4.

**Remark**

It is beyond the scope of this paper to attempt to derive a more realistic expression for the emission current from a conical or cuspidal emitter. The significance of equation (3.3) is that, even in a situation of extreme singularity, the simple model underlying equation (3.2) provides the base for the data collapsing observed experimentally. Note that the calculation has produced a fundamental scale for the energy, *φ*, and a fundamental scale for the length $\Lambda_{\varphi }≐\sqrt{h^{2}/2m\varphi }$. Typical values [9] for these parameters are *φ*≈4.5 *eV* and *Λ*_*φ*_≈0.6 *nm*. *Λ*_*φ*_ is the De Broglie wavelength corresponding to the maximum barrier height *φ*. Finally, we note that the case corresponding to a planar electron emitter in front of a planar counterelectrode [13–15] is also covered by the scaling law when the suitable value λ_1_=1 is inserted.

### (b) Rounded tips: analytical potential

Realistic tips—such as those described by the sphere-on-the-cone, hyperboloid and parabolic models—might have some rounding, characterized by a spatial scale that we called *a* in §2 and is absent in the conical geometry. Accordingly, as shown in §2, the rounding produces for all of them a potential with leading *linear* term within the tunnelling barrier. The scaling of the potential with *d* depends on whether the planar counterelectrode providing one boundary condition is distant (*Φ*(*z*)∼(*a*/*d*)^λ_1_^⋅*Φ*_*d*_⋅*z*/*a*) or near (*Φ*(*z*)∼*Φ*_*d*_⋅*z*/*d*). In both cases, the integrals in equation (3.2) can also be computed in terms of elementary functions [11,13] and the resulting scaling laws write

3.4 $$\begin{array}{rl} |J^{\mathrm{near}}(\Phi_{d},d)| & =J^{\mathrm{near}}\left(\frac{e\Phi_{d}}{\varphi }\cdot {\left(\frac{d}{\Lambda_{\varphi }}\right)}^{-1}\right)\\ |J^{\mathrm{distant}}(\Phi_{d},d)| & =J^{\mathrm{distant}}\left(\frac{e\Phi_{d}}{\varphi }\cdot {\left(\frac{d}{a}\right)}^{-\lambda_{1}}\right)\\ \text{and}\;J^{\mathrm{distant},\;\mathrm{near}}(x) & =a_{1}^{\mathrm{distant},\;\mathrm{near}}\cdot x^{2}\cdot e^{-a_{2}^{\mathrm{distant},\;\mathrm{near}}\cdot x^{-1}}. \end{array}\}$$

Note that for near planes, the scale *a* cancels out from the problem and only one fundamental scale remains, namely *Λ*_*φ*_. By contrast, for distant planes, the problem has two fundamental scales: *a*—originating from the geometry of the tip—and *Λ*_*φ*_—originating within the quantum mechanics of the tunnelling process. The dimensionless constants *a*_1_ and *a*_2_ depend on the geometry, on natural constants and, in the case of distant planes, on *a*/*Λ*_*φ*_.

### (c) Proposal for a unified approach and relationship with direct tunnelling

If we introduce the barrier length $\Delta ≐|z_{2}-z_{1}|$, the tunnelling current densities for the non-analytic potential (equations (B3) and (B4)) and for the linear potential (equation (B7)) can be unified into one single expression in the variables *φ* (the barrier height) and Δ:

3.5 $$J(\Delta ,\varphi )=b_{1}\frac{e\varphi }{h}\cdot \frac{1}{\Delta^{2}}\cdot e^{-b_{2}(\Delta /\Lambda_{\varphi })},$$

where *b*_1_ and *b*_2_ are some numbers that take into account the exact shape of the potential within the tunnelling barrier. Note that the exponential (leading) term in equation (3.5) appears also in models of direct quantum tunnelling, the process underlying STM (see eqn. (25) in ref. [1]), where Δ is given by the tip-to-surface distance. Therefore, it seems that equation (3.5)—at least its exponential factor—is a general motive in tunnelling phenomena. What distinguishes direct tunnelling from electric-field-assisted tunnelling—the process described in this paper—is the functional dependence of *J* on *Φ*_*d*_,*d*. In the case of electric-assisted tunnelling, the scaling hypothesis imposes the special functional dependence of Δ on *Φ*_*d*_,*d*, i.e. Δ=Δ(*Φ*_*d*_⋅*d*^−λ^). The current density behaves accordingly and the functional dependence is reduced to one single variable. In direct tunnelling, this reduction to one single variable is not realized (see eqn (25) in ref. [1] and ref. [3]). We also mention here that the spin of the electrons is a further variable that appears in direct (STM) tunnelling phenomena [21]. It would be interesting to study whether the spin also plays a role in electric-field-assisted tunnelling and its scaling properties.

## 4. Experimental and numerical evidence of the scaling hypothesis

### (a) Experimental evidence

#### (i) *I*–*V* curves

**Figure 6.**
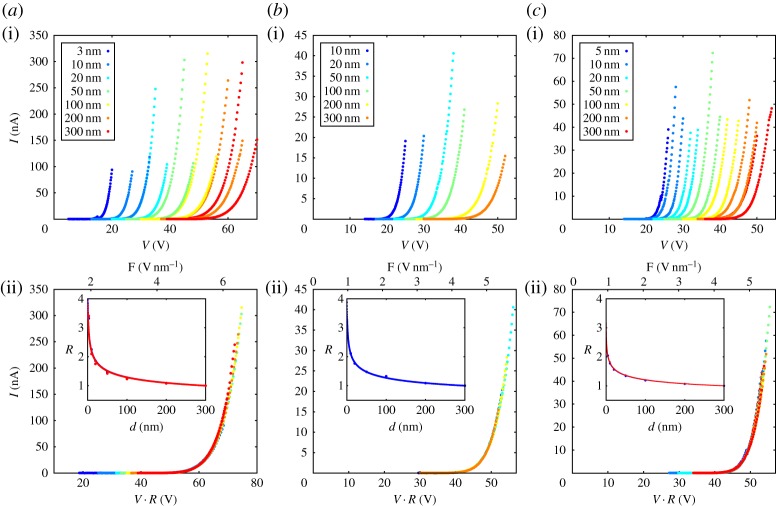
(*a*–*c*)(i) Family of *I*–*V* curves in the range of *d*=3–300 nm. (*a*–*c*)(ii) The curves on the top are made to collapse onto a reference curve (chosen arbitrarily as the one corresponding to *d*=300 *nm*) by multiplying the voltage with a number *R*(*d*), plotted in the insets on the bottom. The continuous curves through *R*(*d*) in the insets are power laws ∝*d*^−λ^, with λ≈0.2±0.05. The alternative horizontal scale in the bottom figure gives the electric field *F* at the apex, as derived in ref. [9].

The first observation of the scaling behaviour is contained in the experimental data reported in ref. [9]. We reproduce part of these data in figure 6*a*(i,ii), which also contains further experimental data (*b*(i,ii) and *c*(i,ii)). Most remarkably, these further data, taken with different tips, strongly support the collapsing behaviour reported in ref. [9]. We conclude that the scaling behaviour is a general feature of electric-field-assisted tunnelling. Let us discuss now some details of the data in figure 6. The top panel of the figure shows a set of current (*I*) versus voltage (*V*) curves taken for three different tips (*a*,*b*,*c*) at different distances from a doped Si(111)-single crystal surface used as counterelectrode. Experimental details about the measurements are reported in ref. [9]. We recall that our tips are fabricated starting with a tungsten wire with a few millimetres length and 250 μ*m* diameter. The last few hundreds of micrometres close to one end of the wire are etched electrochemically to assume a cuspidal profile which, in the final few micrometres towards the apex, resembles very much a cone with a full angle of aperture between 6° and 12°. Electron microscope imaging of the tip reveals a rounding of the tip towards its apex. The rounding varies between 5 and 30 nm, depending on the details of the tip preparation in ultra-high vacuum [9]. The counterelectrode is typically a W(110) or a Si(111) single-crystal surface: it turned out that low-noise *I*–*V* characteristics are favoured by the use of typically very flat Si(111)-surfaces. The family of curves in the top of the figure collapses onto one single reference curve (figure 6*a*–*c*(ii)) when the voltage is multiplied by a *d*-dependent factor *R*(*d*), which is well described by a power law of the type ∼*d*^−λ^, with λ∼0.2. The analysis of the data presented in figure 6 demonstrates that *I* is a function of *V* ⋅*d*^−λ^, i.e. $I=I(V\cdot d^{-\lambda })$. This is the essential point of the scaling hypothesis proposed in §3. Furthermore, the experimentally observed values for the exponent λ fall in the range of values expected for λ_1_ by equations (2.2) and (2.4). We recall that the scaling hypothesis in §3 refers to the current *density* while experiments measure the total tunnelling *current*. Yet, the scaling hypothesis is realized for the current as well. This points to the fact that the details of the area on the apex where the electrons originate from (uniformity of emission, variable size of the area of emission with *d* and *V*) are not relevant for the scaling behaviour. In other words, the tunnelling process is dominated by the exponential function over any multiplicative, probably non-exponential prefactor.

#### (ii) *V* –*d* curves

**Figure 7.**
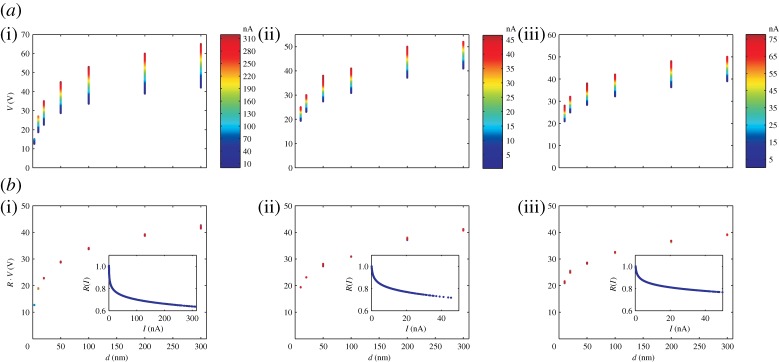
*a*(i–iii) Family of *V* –*d* curves (data taken from figure 6), each curve corresponding to a given current (colour coded along the vertical bar). *b*(i–iii) The family of curves is collapsed onto one single reference curve by multiplying the voltage with a number *R*(*I*), plotted in the inset.

The scaling equation $I=I(V\cdot d^{-\lambda })$ can be inverted to yield $V=I^{\left(-1\right)}(I)\cdot d^{\lambda }$, where $I^{\left(-1\right)}$ indicates the inverse scaling function. Accordingly, if we plot the experimental data of figure 6 in a *V* –*d* diagram, a family of *V* –*d* curves appears (figure 7*a*(i-iii)). When *V* is multiplied by the scaling factor $R(I)≐1/I^{\left(-1\right)}(I)$ (see insets in figure 7*b*(i–iii)), the family of *V* –*d* curves collapses onto one single reference curve, behaving as *d*^λ^, with the exponent being the same entering the insets of figure 6. We find that the factor *R*(*I*) closely follows a curve of the type ≈*I*^−μ^, with a very small value for μ [9]. It follows that $1/I^{\left(-1\right)}(I)\approx I^{-\mu }$ and therefore $I(x)\approx x^{1/\mu }$. We note that, in the range available experimentally, this scaling function describes properly the experimental results but is unusual in tunnelling phenomena. On the other side (see ref. [18], pp. 40–41 for a complete discussion), a very small critical exponent might be the sign of a peculiar singular behaviour, like, for example, a logarithmic singularity. In fact, for small μ and moderate currents, one has $I^{-\mu }\approx 1-\mu \ln I$, and thus $1/I^{\left(-1\right)}(I)\approx 1-\mu \ln I$. Inverting the latter, we obtain $I(x)\approx e^{-1/\mu x}$. This is the scaling function predicted by equation (3.4) and, in the available range of experimental values of *I*, it appears to describe as properly as the scaling function *x*^1/μ^ the bulk of the experimental data. Note, however, that on the basis of the experimental data alone, it is impossible to discriminate between the scaling functions *x*^1/μ^ and *e*^−1/μ*x*^. The two functions could hypothetically be distinguished in the limit of *x* going to infinity. However, this limit corresponds to very large currents, while a maximal current of only a few microamperes can be driven through realistic tips without destroying them. Note also that there are still some details of the reference graphs in figures 6 and 7 which are not properly covered by either scaling functions.

#### (iii) Deviations from scaling

**Figure 8.**
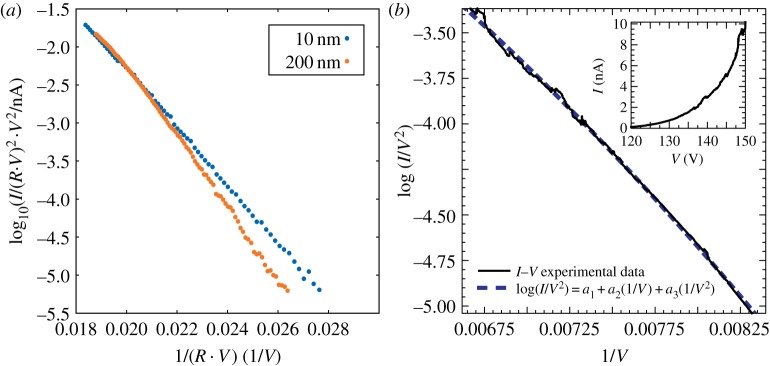
(*a*) Two *I*–*V* curves selected from figure 6 are plotted in a $\log_{10}(I/V^{2})$ versus 1/*V* plot, showing a small downward curvature and a deviation from scaling at low currents. (*b*) *I*–*V* curve measured at *d*=175 *nm* in a junction with *W*(110) single crystal plane as a counterelectrode. The tip used in this experiment is the one described in figure 9. The continuous curve is a fit with a function of the type $\log_{10}(I/V^{2})=a_{1}+a_{2}\cdot 1/V+a_{3}\cdot 1/V^{2}$, *a*_*i*_ being the fitted parameters. Inset: the *I*–*V* data in a linear plot. (Online version in colour.)

In order to enhance these details, we single out two typical *I*–*V* curves within a so-called Fowler–Nordheim [13] plot of $\log(I/V^{2})$ versus 1/*V* (figure 8*a*). Although the scaling function of equation (3.4) predicts a strict linearity of the graphs in this kind of plot, we observe a systematic downward curvature of the graphs, which is more pronounced at larger distances. The observed curvature represents a deviation from the scaling function predicted by equation (3.4), while the separation of the two curves on figure 8*a* for small voltages means a slight deviation from collapsing towards smaller currents. In the following, we offer some arguments that might account for these observations.

The scaling function for a non-analytical potential implies naturally the experimentally observed downward curvature, but it cannot be applied straightforwardly to real tips, which have necessarily a rounded apex. A simple way of avoiding the unphysical non-analyticity by *simultaneously* keeping its welcome nonlinearity is to introduce a finite cut-off length *r* into the cone solution by substituting the power-law (*z*/*d*)^λ_1_^ with its Taylor series at a finite distance *r* from the tip apex. Here, *r* might be considered a measure for the radius of curvature. This yields

4.1 $$\sqrt{\varphi -eV\cdot A\cdot {\left(\frac{z}{d}\right)}^{\lambda_{1}}}\to \sqrt{\varphi -\Phi (r)-eV\cdot \lambda_{1}\cdot A\cdot {\left(\frac{r}{d}\right)}^{\lambda_{1}}\left[\frac{z-r}{r}-\frac{1-\lambda_{1}}{2}\frac{{\left(z-r\right)}^{2}}{r^{2}}+\cdots \right]},$$

where *A* being a dimensionless factor (see appendix A). Note that a similar quadratic correction to the linear term can also be found using the sphere-on-the-cone or the hyperboloidal models for a rounded tip, but the mathematics entailed by the physically plausible cut-off approximation is more transparent. We also observe that this approximation introduces a linear and a quadratic term into the potential within the tunnelling barrier, but the presence of the quadratic term does not break the scaling invariance. The simplest way of taking the quadratic term into account in the calculation of the tunnelling current density is to use the proposed unifying equation, which implies finding the value of *z* for which the argument of the square root in equation (4.1) vanishes (*Φ*(*r*) is set for convenience to zero by modifying the boundary condition so that the zero of the potential is at *z*=*r*). Mathematically speaking, this means solving a quadratic equation. In the range of *V* for which the equation has a solution, we approximately find

4.2 $$\Delta \approx r\cdot \frac{\varphi }{\lambda_{1}eVA{\left(r/d\right)}^{\lambda_{1}}}+r\cdot \frac{1-\lambda_{1}}{4}{\left(\frac{\varphi }{\lambda_{1}eVA{\left(r/d\right)}^{\lambda_{1}}}\right)}^{2}$$

and accordingly

4.3 $$J(V,d)\approx \exp\left[-a_{1}\frac{r\cdot \varphi /\lambda_{1}eVA{\left(r/d\right)}^{\lambda_{1}}+r\cdot (1-\lambda_{1})/4{\left(\varphi /\lambda_{1}eVA{\left(r/d\right)}^{\lambda_{1}}\right)}^{2}}{\Lambda }\right].$$

The quadratic part of the potential, which mimics the upward curvature of the potential energy entailed by the original power law, increases the tunnelling width above the value obtained with the linear term only and introduces an extra term which is responsible for the downward curvature observed in the experimental Fowler–Nordheim plots (see figure 8*a* and ref. [9]). Note that equation (4.3) still obeys the scaling hypothesis which is apparently ‘robust’ also with respect to quadratic corrections of the potential. However, the appearance of the non-leading power (*r*/*d*)^2λ_1_^ in the expression for the tunnel width suggests that, ultimately, a realistic expression for the current density will need the use of other non-leading powers of *r*/*d* as well. But as soon as we allow non-leading powers of the type (*r*/*d*)^λ_*k*_^, the scaling behaviour is broken and deviation from collapsing is expected (and indeed observed, see ref. [9] and figure 8*a*). We conclude that a scaling function of the type $J(x)=a_{1}\;e^{-a_{2}/x-a_{3}/x^{2}}$, which generalizes the expression obtained in equation (4.3) for a nonlinear potential, might be useful to interpolate experimental data that show a downward curvature and small deviations from scaling in Fowler–Nordheim plots. An example of such a procedure is reported here in figure 8*b*. A similar proposal was also put forward in ref. [22] on the basis of numerical results (see also ref. [23] for a recent discussion on the curvature problem).

### (b) Numerical evidence

The principle of Saint-Venant [24] implies a further interesting scaling symmetry of the electric-field-assisted tunnelling junction. In fact, this principle can be used to extend the validity of the conical solution, equation (2.2), to real tips, supposing that they can be viewed as a ‘cone with a rounded apex’. According to the principle of Saint-Venant, if the rounding of the cone singularity is local enough—say limited to a scale length *ϱ*, which does not need to be atomic, describing the ‘radius of curvature’—then the conical solution, equation (2.2), can be used in the range *ϱ*≪*z*≪*d* as well (the origin of the *z*-axis being, as usual, the apex of the tip):

4.4 $$\Phi (z)∼\Phi_{d}\cdot {\left(\frac{z}{d}\right)}^{\lambda_{1}},\;a\ll z\ll d.$$

**Figure 9.**
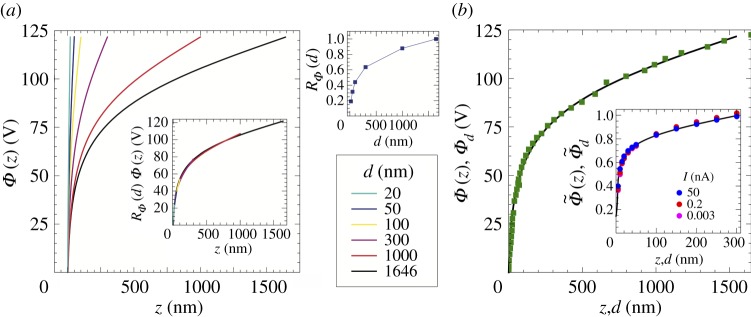
(*a*) Potential profile *Φ*(*z*) along the tip axis, for a given distance *d*. The tip used in this simulation is a hyperboloidal model of a ‘real’ tip (*a*= 1528 nm, *ω*_0_=11°). The planar counterelectrode is moved between *d*= 20 nm and *d*=1646 nm. All profiles are made to approximately collapse onto the reference curve (*d*=1646 nm) when the potential *Φ*(*z*) is multiplied by *R*_*Φ*_(*d*) (see inset). *R*_*Φ*_(*d*) is shown in the top right corner of the figure. Note that both *R*_*Φ*_(*d*) and the scaled profile in the inset behave as a power law, with a power-law exponent of about 0.3. This value is larger than the one of about 0.21 expected from *ω*_0_=11°. We point out that the value of 0.21 is an asymptotic value expected when the planar counterelectrode is much farther away than the confocal plane. In the present simulation, *d* is smaller or in the range of the confocal plane distance so that we might not have reached yet the true power law range. In fact, we have performed computations with *d*=6000 nm (not shown) and the exponent is seen to converge towards the analytical value of 0.21. We note, however, that the collapsing of profiles is realized also in this non-asymptotic range, showing that the scaling property itself is a robust one. (*b*) Scaling of *Φ*(*z*) and *Φ*_*d*_(*d*). The continuous line is the potential profile obtained as described above, for *d*=1646 nm. The full squares are *Φ*_*d*_(*d*) data points obtained at a given current, the current of 150 pA having being chosen so that the *Φ*_*d*_(*d*) curve almost lies onto the *Φ*(*z*) graph, without need of a rescaling factor. Inset: *Φ*_*d*_(*d*) data points obtained in a junction with Si(111) at selected currents, given in the legend. In the inset, data are rescaled so that they fall onto the same power law. The potential profile computed for *d*=300 nm (continuous curve) can also be rescaled onto the same curve as the *Φ*_*d*_(*d*) data.

This equation is valid for any real tip shape with arbitrary but sufficiently localized rounding, provided that asymptotically away from the rounded apex the conical shape is recovered. Note that the region of validity of this equation makes it irrelevant for the field emission process, which occurs at positive *z*≪*ϱ*, so that the discussion of this paragraph refers only to a property of the electrostatic potential. Equation (4.4) provides a further scaling law which can be tested numerically by verifying, e.g., the collapsing of the family of *Φ*(*z*,*d*)-curves taken for different *d* onto one single curve, when *Φ*(*z*) is multiplied by a suitable *R*_*Φ*_(*d*)∼*d*^λ_1_^ (figure 9*a*). The latter is inversely proportional to *R*(*d*) introduced previously (figure 6), i.e. *R*_*Φ*_(*d*)∼1/*R*(*d*). For the computation of the potential, we have fitted a hyperboloid of revolution onto the electron microscope micrograph of the tip used for the taking of the experimental *V* –*d*-curves. Subsequently, we have used a numerical routine [25] to compute *Φ*(*z*) in the presence of a plane placed at a well-defined (large) fixed distance in front of the hyperboloidal tip. A further consequence of equation (4.4) is that an experiment where *d* is changed and *Φ*_*d*_ is adjusted so that the tunnelling current (i.e. the potential within the tunnelling barrier) is kept constant yields *Φ*_*d*_∼*d*^λ^, i.e. the same power-law dependence that one expects for the *z*-dependence of the potential *Φ* itself. In other words, we expect that all experimental *Φ*_*d*_−*d* graphs (i) can be made to collapse onto themselves (figure 7 and inset of figure 9*b*) and (ii) they can be collapsed onto a *Φ*(*z*) profile, provided that one is not too close to *z*=*d* or *z*=0 (figure 9*b*).

## Appendix A. Electrostatics

We will consider tips (‘needles’) with ‘realistic’ but highly symmetric geometries. The most important symmetry, common to all tips, is the rotational symmetry with respect to the tip axis. One speaks of axially symmetric Laplace problem because the sought for potential will be independent on the rotational angle *ϕ* (figure 1) and that part of the Laplace operator which contains derivatives with respect to *ϕ* can be neglected from the beginning. A further symmetry is imposed on the electrostatic problem: the surface of the tip has a particular geometry along which, when suitable, typically *curvilinear* coordinates $\tilde{x}$ and $\tilde{y}$ are introduced and one of them (say, $\tilde{y}$) assumes a fixed value ${\tilde{y}}_{0}$. One of the boundary conditions reads therefore: $\Phi (\tilde{x},{\tilde{y}}_{0})=0$ $\forall \tilde{x}$. In view of the need to fulfil this boundary condition, it is suitable to introduce a *separation Ansatz* $\Phi (\tilde{x},\tilde{y})=X(\tilde{x})\cdot Y(\tilde{y})$ for solving the associated Laplace equation, yielding

A 1 $$\frac{{△}_{\tilde{x}}X(\tilde{x})}{X(\tilde{x})}=-\frac{{△}_{\tilde{y}}Y(\tilde{y})}{Y(\tilde{Y})},$$

where ${△}_{\tilde{x},\tilde{y}}$ denote the Laplace operators in the variables $\tilde{x}$ and $\tilde{y}$, respectively. Because the two sides of the equation depend on two different variables, the problem reduces to two ordinary differential equations

A 2 $$\begin{array}{rl} & \frac{{△}_{\tilde{x}}X(\tilde{x})}{X(\tilde{x})}=\lambda \cdot (\lambda +1)\\ \text{and}\; & \frac{{△}_{\tilde{y}}Y(\tilde{y})}{Y(\tilde{Y})}=-\lambda \cdot (\lambda +1), \end{array}\}$$

where λ⋅(λ+1) is a separation constant to be determined by implementing the boundary conditions. Equations (A 2), in general, have two linear independent solutions $X_{\lambda }^{\left(1,2\right)}(\tilde{x})$ and $Y_{\lambda }^{\left(1,2\right)}(\tilde{y})$. The boundary condition on the tip results in two eigenvalue problems for the separation constant λ(λ+1):

A 3 $$\begin{array}{rl} & Y_{\lambda }^{\left(1\right)}({\tilde{y}}_{0})=0\\ \text{and}\; & Y_{\lambda }^{\left(2\right)}({\tilde{y}}_{0})=0. \end{array}\}$$

The solution of equation (A 3) is a countable set of values λ_*h*_ and λ_*k*_ with *h*=1,2,… and *k*=1,2,…, which we can order starting from the smaller one. By superposition, the general solution of the Laplace equation fulfiling the boundary condition on the tip reads

A 4 $$\begin{array}{rl} \Phi (\tilde{x},\tilde{y},\phi ) & =\sum_{\lambda_{h}}[A_{h}^{\left(1\right)}\cdot X_{h}^{\left(1\right)}(\tilde{x})+A_{h}^{\left(2\right)}\cdot X_{h}^{\left(2\right)}(\tilde{x})]Y_{h}^{\left(1\right)}(\tilde{y})\\ & \;+\sum_{\lambda_{k}}[A_{k}^{\left(1\right)}\cdot X_{k}^{\left(1\right)}(\tilde{x})+A_{k}^{\left(2\right)}\cdot X_{k}^{\left(2\right)}(\tilde{x})]Y_{k}^{\left(2\right)}(\tilde{y}), \end{array}$$

where the coefficients $A_{h,k}^{\left(1,2\right)}$ in general depend on *d*,*Φ*_*d*_ and are determined by the boundary condition on the plane. The conducting plane is generally described by an equation of the type $\tilde{x}=P(\tilde{y},d)$ and does not necessarily coincide with a surface of the type $\tilde{y}={\tilde{y}}_{0}$. By implementing the boundary condition on the plane, we obtain the following condition:

A 5 $$\begin{array}{rl} \Phi_{d} & =\sum_{\lambda_{h}}[A_{h}^{\left(1\right)}\cdot X_{h}^{\left(1\right)}(P(\tilde{y},d))+A_{h}^{\left(2\right)}\cdot X_{h}^{\left(2\right)}(P(\tilde{y},d))]Y_{h}^{\left(1\right)}(\tilde{y})\\ & \;+\sum_{\lambda_{k}}[A_{k}^{\left(1\right)}\cdot X_{k}^{\left(1\right)}(P(\tilde{y},d))+A_{k}^{\left(2\right)}\cdot X_{k}^{\left(2\right)}(P(\tilde{y},d))]Y_{k}^{\left(2\right)}(\tilde{y}), \end{array}$$

which must be fulfiled for any $\tilde{y}$. Consequently, an infinite set of equations for the infinite number of coefficients $A_{h,k}^{\left(1,2\right)}$ is obtained.

*Scaling hypothesis*. The analysis of the boundary condition equation (A 5) determines the scaling properties of the potential with *d* and *Φ*_*d*_. We note that the scaling property with *Φ*_*d*_ follows directly from the fact that equation (A 5) must hold for any value of *d* and $\tilde{y}$, such that each term in the sum must scale with *Φ*_*d*_ as

A 6 $$A_{h,k}^{\left(1,2\right)}(d,\Phi_{d})∼\Phi_{d}\cdot A_{h,k}^{\left(1,2\right)}(d,1).$$

The scaling behaviour of the potential with *d* is determined by the specific geometry of the considered problem.

**(a) Conical tip**

*Differential equations*. We introduce spherical coordinates (*r*,*ϑ*,*ϕ*) with respect to the origin of the coordinate system [26] (figure 2), such that the surface of the cone is a surface of constant polar angle *ϑ*=*ϑ*_0_. With the separation Ansatz *Φ*(*x*,*y*,*z*)=*R*(*r*)⋅*Θ*(*ϑ*), we obtain the following Laplace equation:

A 7 $$\begin{array}{ll} & r^{2}\frac{R^{\prime \prime }(r)}{R(r)}+2r\frac{R^{\prime }(r)}{R(r)}=-\frac{1}{\Theta (\vartheta )\sin\vartheta }\frac{\partial }{\partial \vartheta }\left(\sin\vartheta \frac{\partial }{\partial \vartheta }\Theta (\vartheta )\right)\\ & \;⟺\\ & r^{2}R^{\prime \prime }(r)+2rR^{\prime }(r)-\lambda \cdot (\lambda +1)R(r)=0\\ \text{and}\; & \frac{1}{\sin\vartheta }\frac{\partial }{\partial \vartheta }\left(\sin\vartheta \frac{\partial }{\partial \vartheta }\Theta (\vartheta )\right)+\lambda \cdot (\lambda +1)\Theta (\vartheta )=0, \end{array}\}$$

the lowest one being the Legendre differential equation.

*General solution*. For any value of λ, the linearly independent solutions of equation (A 7) are

A 8 $$\begin{array}{rl} & R_{\lambda }^{\left(1\right)}(r)=r^{\lambda },\;R_{\lambda }^{\left(2\right)}(r)=\frac{1}{r^{\lambda +1}}\\ \text{and}\; & \Theta^{\left(1\right)}(\cos\vartheta )=P_{\lambda }(\cos\vartheta ),\;\Theta^{\left(2\right)}(\cos\vartheta )=Q_{\lambda }(\cos\vartheta ), \end{array}\}$$

where $P_{\lambda }(\cos\vartheta )$ and $Q_{\lambda }(\cos\vartheta )$ denote the Legendre functions of the first and second kind [27]. Some of these functions are, however, not suitable as solutions of our conical tip problem. In fact, for non-integer values of λ, the Legendre function of the first kind is regular for all angles 0≤*ϑ*<*π*, but it is singular for *ϑ*=*π*. However, this singularity is excluded from *Ω* so that $P_{\lambda }(\cos\vartheta )$ can be admitted as solution. Legendre functions of the second kind, however, are singular for both *ϑ*=0 and *ϑ*=*π*, such that we must exclude them, as the line *ϑ*=0 is part of *Ω*. Similarly, *R*^(2)^(*r*) has a singularity at *r*=0 and therefore cannot enter the general solution of our problem. The general solution of equation (A 7) reads, therefore,

A 9 $$\Phi (x,y,z)=\sum_{\lambda }A_{\lambda }(\Phi_{d},d)\cdot r^{\lambda }\cdot P_{\lambda }(\cos\vartheta ),$$

where the sum runs over a countable set of values λ, which is determined by the boundary condition on the cone.

*The eigenvalue problem: boundary condition on the tip*. The allowed values for the separation constant λ are obtained by implementing the boundary condition on the cone

A 10 $$P_{\lambda }(\cos\vartheta_{0})=0.$$

The above equation has an infinite countable number of solutions λ_*k*_(*ϑ*_0_), *k*=1,2,3,…, which can be ordered with increasing magnitude and have been calculated as a function of *ϑ*_0_, for example, in ref. [17]. For sharp cones (*ϑ*_0_≈*π*), the smallest index λ_1_ can be written approximately as a function of the the full angle of aperture of the cone $\omega_{0}≐2\pi -2\vartheta_{0}$

A 11 $$\lambda_{1}(\omega_{0})⋍\frac{1}{\left[2\ln(2/\omega_{0})\right]}.$$

In summary, the general solution of the electrostatic problem pertaining to a conical tip reads

A 12 $$\Phi (r,\vartheta )=\sum_{k}A_{k}(d,\Phi_{d})r^{\lambda_{k}}P_{\lambda_{k}}(\cos\vartheta ).$$

*Scaling behaviour: the boundary condition on the plane.* The coefficients *A*_*k*_ are uniquely determined by imposing the boundary condition on the plane placed in front of the tip, defined by the equation rcos⁡ϑ=d with 0≤*ϑ*≤*π*/2:

A 13 $$\sum_{k}A_{k}(d,\Phi_{d}){\left[\frac{d}{\cos\vartheta }\right]}^{\lambda_{k}}P_{\lambda_{k}}(\cos\vartheta )=\Phi_{d}.$$

Because equation (A 13) must hold for any value of the parameter *d*, the *d*-dependence must cancel out from each term on the left-hand side. This requirement produces the scaling law for the parameter *d*, which combined with equation (A 6) yields

A 14 $$A_{k}(d,\Phi_{d})=A_{k}(1,1)\cdot \Phi_{d}\cdot d^{-\lambda_{k}}.$$

*The potential in the vicinity of the tip apex*. The infinite set of constants *A*_*k*_(1,1) is determined by requiring that equation (A 13) is fulfiled for 0≤*ϑ*≤*π*/2. In principle, by multiplying both sides by, for example, $P_{\lambda_{q}}(\cos\vartheta )$ and integrating over the range 0≤*ϑ*≤*π*/2, one can transform this boundary condition into a system of an infinite number of coupled linear equations for the infinite number of constants *A*_*k*_(1,1). Unfortunately, finding *A*_*k*_(1,1) is complicated by the fact that the functions of cos⁡ϑ appearing in the sum do not obey simple orthonormality relations in the range 0≤*ϑ*≤*π*/2, so that the diagonalization of the resulting matrix is a cumbersome numerical problem which can be solved only approximately. It is, however, not our purpose to find the entire set (or even a large number) of the constants *A*_*k*_(1,1). In fact, of relevance to the discussion of §3 is only the potential close to the tip apex and along the tip axis *z*, which is given by the leading term in equation (A 12). Provided that the term equation (A 12) corresponding to λ_1_ is not vanishing, we have

A 15 $$\Phi (z)∼\Phi_{d}\cdot d^{-\lambda_{1}}z^{\lambda_{1}}.$$

The non-vanishing of this term can be proved using the maximum principle for harmonic functions, which states that any harmonic function on a domain *Ω* with boundary ∂*Ω* takes its maximum and minimum values necessarily on the boundary. This implies that *A*_1_≠0: otherwise, the leading term would be the one corresponding to λ_2_, but *P*_λ_2__ also vanishes again for *ϑ*<*ϑ*_0_, thus violating the maximum principle for harmonic functions. Note that this expression for the potential is valid for any distance *d*, as, in the vicinity of a cone-singularity, *r* can be made arbitrarily small.

**(b) Sphere on the cone**

This is a slight modification of the conical problem but allows an exact model of a rounding of the tip apex, which is expected for realistic tips.

*General solution*. The cone acts as a post supporting a small sphere of radius *a*. The sphere replaces the point singularity at the apex of the cone. For the computations in this particular section, it is convenient to set the origin of the coordinate system at the centre of the sphere, such that we write (*x*,*y*,*z*′=*z*+*a*), where *z* is measured from the tip apex (figure 3). In this coordinate system, the position of the plane is given by *z*′_*P*_=*d*+*a*. The general solution of this axially symmetric problem follows the same lines as in the previous subsection. In this case, the general solution reads

A 16 $$\Phi (r,\vartheta )=\sum_{k}A_{k}(z_{P}^{\prime },\Phi_{d})\left[{\left(\frac{r}{a}\right)}^{\lambda_{k}}-{\left(\frac{a}{r}\right)}^{\lambda_{k}+1}\right]P_{\lambda_{k}}(\cos\vartheta ),$$

where λ_*k*_(*ϑ*_0_), *k*=1,2,3,… are the solutions of equation (A 10).

**(i) Scaling behaviour: distant planes**

The boundary condition on the plane requires

A 17 $$\Phi_{d}=\sum_{k}A_{k}(z_{P}^{\prime },\Phi_{d})\left[{\left(\frac{z_{P}^{\prime }}{a\cos\vartheta }\right)}^{\lambda_{k}}-{\left(\frac{a\cos\vartheta }{z_{P}^{\prime }}\right)}^{\lambda_{k}+1}\right]P_{\lambda_{k}}(\cos\vartheta )$$

for all 0≤*ϑ*≤*π*/2. In order to find the scaling behaviour with *Φ*_*d*_,*z*′_P_, however, it is sufficient to consider the boundary condition along the tip axis and thus we set *ϑ*=0 in equation (A 17). For *z*′_P_≫*a*, equation (A 17) reduces to

A 18 $$\sum_{k}A_{k}(z_{P}^{\prime }\gg a,\Phi_{d}){\left(\frac{z_{P}^{\prime }}{a}\right)}^{\lambda_{k}}P_{\lambda_{k}}(1)=\Phi_{d},$$

wherefore the following scaling relation holds

A 19 $$A_{k}(z_{P}^{\prime }\gg a,\Phi_{d})∼\Phi_{d}\cdot {\left(\frac{a}{z_{P}^{\prime }}\right)}^{\lambda_{k}}.$$

Accordingly, the leading order term of the potential in the vicinity of the tip apex and along the tip axis reads

A 20 $$\Phi (z^{\prime })∼\Phi_{d}\cdot {\left(\frac{a}{z_{P}^{\prime }}\right)}^{\lambda_{1}}\frac{\left(z^{\prime }-a\right)}{a}.$$

When written in terms of the coordinates *x*,*y*,*z* on the tip apex, equation (A 20) recovers equation (2.4) exactly, where we have exploited the fact that *z*′_P_≈*d*, which holds for distant planes.

**(ii) Scaling behaviour: near planes.**

As described above, it is sufficient to set *ϑ*=0 in equation (A 17). For planes close to the tip, (*z*′_P_−*a*)/*a*≪1, wherefore

A 21 $$\left[{\left(\frac{z_{P}^{\prime }}{a}\right)}^{\lambda_{k}}-{\left(\frac{a}{z_{P}^{\prime }}\right)}^{\lambda_{k}+1}\right]∼\frac{z_{P}^{\prime }-a}{a}$$

to leading order. From equation (A 21), we deduce the scaling behaviour

A 22 $$A_{k}\left(\frac{a}{z_{P}^{\prime }-a}\ll 1,\Phi_{d}\right)∼\Phi_{d}\cdot \left(\frac{z_{P}^{\prime }-a}{a}\right).$$

The leading order term of the potential along the *z*′-axis and in the vicinity of the tip apex is thus given by

A 23 $$\Phi (z^{\prime })∼\Phi_{d}\cdot \frac{z^{\prime }-a}{z_{P}^{\prime }-a},$$

which is exactly equation (2.4) in terms of the coordinates (*x*,*y*,*z*).

**(c) Hyperboloid of revolution**

*Differential equations and general solution.* The tip surface is modelled as a hyperboloid of revolution, obtained by rotating a hyperbola around the axis containing its focus with coordinates *x*=*y*=0 and *z*′=−*a* (figure 3). In order to implement the boundary condition on the tip surface, it is convenient to introduce the prolate-spheroidal coordinates (μ,*ν*,*ϕ*) [28], with μ∈[0,∞[, *ν*∈[0,*π*], and *ϕ*∈[0,2*π*], defined through

A 24 $$\begin{array}{rl} & x=a\sinh\mu \sin\nu \cos\phi ,\\ & y=a\sinh\mu \sin\nu \sin\phi \\ \text{and}\; & z^{\prime }=a\cosh\mu \cos\nu . \end{array}\}$$

Surfaces of constant μ are ellipsoids of revolution with foci at *z*′=±*a*, where the limiting case μ=0 is a straight line connecting the foci. Surfaces of constant *ν* are hyperboloids with focus at +*a* for *ν*∈[0,*π*/2] and at −*a* for *ν*∈[*π*/2,*π*]. In particular, the surface of the tip is *ν*=*ν*_0_. The corresponding Laplace equation reads

A 25 $$\frac{1}{a^{2}}\frac{1}{\xi^{2}-\eta^{2}}\left[\frac{\partial }{\partial \xi }(\xi^{2}-1)\frac{\partial }{\partial \xi }+\frac{\partial }{\partial \eta }(1-\eta^{2})\frac{\partial }{\partial \eta }\frac{\xi^{2}-\eta^{2}}{\left(\xi^{2}-1)(1-\eta^{2}\right)}\frac{\partial^{2}}{\partial \phi^{2}}\right]\Phi =0,$$

where we have introduced ξ=cosh⁡μ and η=cos⁡ν. The rotational symmetry of the problem implies that the potential is independent of the angle *ϕ*. With the separation Ansatz *Φ*(*ξ*,*η*)=*Ξ*(*ξ*)*N*(*η*), we obtain the following equations:

A 26 $$\begin{array}{rl} & \frac{\partial }{\partial \xi }\left((1-\xi^{2})\frac{\partial }{\partial \xi }\Xi (\xi )\right)+\lambda (\lambda +1)\Xi (\xi )=0\\ \text{and}\; & \frac{\partial }{\partial \eta }\left((1-\eta^{2})\frac{\partial }{\partial \eta }N(\eta )\right)+\lambda (\lambda +1)N(\eta )=0, \end{array}\}$$

with solution

A 27 $$\Phi (\xi ,\eta )=\sum_{\lambda }[A_{\lambda }P_{\lambda }(\xi )+B_{\lambda }Q_{\lambda }(\xi )]\cdot [C_{\lambda }P_{\lambda }(\eta )+D_{\lambda }Q_{\lambda }(\eta )],$$

where the sum extends over suitable values of λ, determined by implementing the boundary condition on the tip.

**(iii) Scaling behaviour: distant planes (*z*′_P_/*a*≫1)**

In the limit of distant planes, we must exclude terms proportional to *Q*_λ_(*ξ*) in equation (A 27), as they diverge along the line *ν*=0 ($x=y=0,a\leq z^{\prime }<\infty$). The boundary condition $P_{\lambda }(\cos\nu_{0})=0$ is satisfied by the same values λ_*k*_ that we have obtained when considering conical tips. The boundary condition on a distant plane involves finding the behaviour of *P*_λ_*k*__ and *Q*_λ_*k*__ for large arguments. For this purpose, we let μ→∞ and a→0 by keeping r≐a⋅cosh⁡μ constant. To visualize the significance of this limit, consider that in this limit the foci are ‘moved’ towards the origin and acosh⁡μ≈asinh⁡μ, so that constant-*ξ* ellipsoids become constant-*r* spheres. Formally, in the limit μ→∞ with *r*=*aξ*= constant, prolate spheroidal coordinates are in fact the same as spherical coordinates. To appreciate this point, we rewrite the differential equation for *ξ* in terms of *r*

A 28 $$a^{2}\frac{\partial }{\partial r}\left(\left({\left(\frac{r}{a}\right)}^{2}-1\right)\frac{\partial }{\partial r}\Xi_{\lambda_{k}}(r)\right)-\lambda_{k}\Xi_{\lambda_{k}}(r)=0.$$

In the limit a→0, equation (A 28) simplifies to

A 29 $$r^{2}\Xi_{\lambda_{k}}^{\prime \prime }(r)+2r\Xi_{\lambda_{k}}^{\prime }(r)-\lambda_{k}\Xi_{\lambda_{k}}(r)=0,$$

whose solution reads

A 30 $$\Xi_{\lambda_{k}}^{\left(1\right)}(r)=r^{\lambda_{k}}\;\mathrm{A nd}\;\Xi_{\lambda_{k}}^{\left(2\right)}(r)=\frac{1}{r^{\lambda_{k}+1}}.$$

For distant planes, $\Xi^{\left(2\right)}(z_{P}/a\cos\nu )$ becomes negligible and the boundary condition on the plane simplifies to

A 31 $$\Phi_{d}=\sum_{\lambda_{k}}A_{\lambda_{k}}\Xi_{\lambda_{k}}^{\left(1\right)}\left(\frac{z_{P}^{\prime }}{a\cos\nu }\right)\cdot P_{\lambda_{k}}(\cos\nu ),$$

which, in virtue of the asymptotic behaviour of $\Xi_{\lambda_{k}}^{\left(1\right)}(r)$, requires the scaling property

A 32 $$A_{\lambda_{k}}(\Phi_{d},z_{P}^{\prime })∼\Phi_{d}\cdot {\left(\frac{a}{z_{P}^{\prime }}\right)}^{\lambda_{k}}.$$

*Potential close to the apex*. The potential in the vicinity of the tip apex does not contain $Q_{\lambda_{k}}(\cosh\mu )$ which is singular in the interval *z*′∈[−*a*,*a*], corresponding to μ=0. In addition, we point out that in the vicinity of $\cos\nu_{0}$ both $P_{\lambda_{k}}(\cos\nu )$ and $Q_{\lambda_{k}}(\cos\nu )$ are analytic functions having a Taylor expansion starting with a linear term in $\cos\nu -\cos\nu_{0}$, $z_{0}^{\prime }=a\cos\nu_{0}$ being the *z*′-coordinate of the tip apex. Thus, to leading order in (*z*′−*z*′_0_)/*a*, the potential in the vicinity of the hyperboloid apex and along the hyperboloid axis reads

A 33 $$\Phi (z)∼\Phi_{d}\cdot {\left(\frac{a}{z_{P}^{\prime }}\right)}^{\lambda_{1}}\left(\frac{z^{\prime }-z_{0}^{\prime }}{a}\right).$$

This is equivalent to equation (2.4) in terms of the *x*,*y*,*z*-coordinates (recall that $z^{\prime }=z+a\cos\nu_{0}$).

**(iv) Scaling behaviour: near plan (|*z*′_P_−*z*′_0_|/*a*≪1)**

In this case, we must exclude terms proportional to *Q*_λ_(μ) in equation (A 27), as they diverge along the axis μ=0. The boundary condition along the surface of the hyperboloid selects indices λ_*h*_ and λ_*k*_ for which either $P_{\lambda_{h}}(\cos\nu_{0})=0$ (and *B*_λ_*h*__=0) or $Q_{\lambda_{k}}(\cos\nu_{0})=0$ (and *A*_λ_*k*__=0) holds. The general solution then reads

A 34 $$\Phi (\xi ,\eta )=\sum_{\lambda_{h}}A_{\lambda_{h}}P_{\lambda_{h}}(\xi )\cdot P_{\lambda_{h}}(\eta )+\sum_{\lambda_{k}}B_{\lambda_{k}}P_{\lambda_{k}}(\xi )\cdot Q_{\lambda_{k}}(\eta ).$$

We consider the boundary condition on the plane in the case μ=0 and obtain

A 35 $$\begin{array}{rl} \Phi_{d} & =\sum_{\lambda_{k}}A_{\lambda_{k}}P_{\lambda_{k}}(1)\cdot P_{\lambda_{k}}\left(\frac{z_{P}^{\prime }}{a}\right)+\sum_{\lambda_{h}}B_{\lambda_{h}}P_{\lambda }(1)\cdot Q_{\lambda_{h}}\left(\frac{z_{P}^{\prime }}{a}\right). \end{array}$$

Because for *z*′_P_ close to the tip apex both *P*_λ_*h*__ and *Q*_λ_*k*__ can be approximated by linear terms in (*z*′_P_−*z*′_0_)/*a*, equation (A 35) implies the following scaling relations:

A 36 $$A_{\lambda_{h}}∼\Phi_{d}\frac{a}{z_{P}^{\prime }-z_{0}^{\prime }}\;\mathrm{and}\;B_{\lambda_{k}}∼\Phi_{d}\frac{a}{z_{P}^{\prime }-z_{0}^{\prime }}.$$

*Potential near the apex*. To leading order, the potential in the vicinity of the hyperboloid apex and along the hyperboloid axis reads

A 37 $$\Phi (z^{\prime })∼\Phi_{d}\cdot \frac{z^{\prime }-z_{0}^{\prime }}{z_{P}^{\prime }-z_{0}^{\prime }},$$

which is equivalent to equation (2.4) in terms of the coordinates *x*,*y* and *z*.

**(v) Scaling behaviour: confocal plane (*z*′_P_=0)**

This case is special, because the conducting plane coincides with the hyperboloid defined by *ν*=*π*/2, and thus it is confocal to the tip surface. Boundary conditions are therefore homogeneous in the variable *ν*, such that the solution does not depend on μ. This requirement determines uniquely that λ=0 and *B*_0_=0 is the only possible solution. Accordingly, the general solution simplifies to

A 38 $$C_{0}P_{0}(\eta )+D_{0}Q_{0}(\eta )=C_{0}+\frac{D_{0}}{2}\ln\left(\frac{1+\cos\nu }{1-\cos\nu }\right).$$

The constants *C*_0_ and *D*_0_ must be chosen in order to satisfy the boundary conditions *Φ*(*ν*=*ν*_0_)=0 and *Φ*(*ν*=*π*/2)=+*Φ*_*d*_, so that the potential reads, exactly [29]

A 39 $$\Phi (\mu ,\cos\nu ,\phi )=\Phi_{d}-\frac{\Phi_{d}}{\ln((1+\cos\nu_{0})/(1-\cos\nu_{0}))}\cdot \ln\left(\frac{1+\cos\nu }{1-\cos\nu }\right).$$

Close to $z_{0}^{\prime }=a\cos\nu_{0}$ and along the *z*′-axis we have, to leading order in *z*′−*z*′_0_

A 40 $$\begin{array}{rl} \Phi (z^{\prime }) & \approx -\Phi_{d}\frac{2}{\ln((1+\cos\nu_{0})/(1-\cos\nu_{0}))}\cdot \frac{z^{\prime }-z_{0}^{\prime }}{a\cdot (1-\cos^{2}\nu_{0})}\\ & \approx \Phi_{d}\frac{2}{R_{0}}\cdot \frac{1}{\ln(4d/R_{0})}\cdot z. \end{array}$$

Here, we have used the relation $a^{2}\cos^{2}\nu_{0}=d^{2}$ and $\cos\nu_{0}=\sqrt{d/(d+R_{0})}$, *R*_0_≪*d* being the radius of curvature of the hyperboloid. This result shows that the linear dependence on 1/*d* of the potential crosses over to a logarithmic dependence when the planar electrode crosses the confocal plane. This is consistent with the idea that equation (1.1) corresponds to a logarithmic dependence on *d* when λ=0, which we put forward when discussing the paraboloid solution for *d*≫*R*_0_. When the plane becomes a distant one a true power law intervenes.

*A remark*. One might be tempted to use the method just illustrated to solve the electrostatic problem of an infinitely thin semi-infinite needle placed vertical to a conducting plane [30]. In fact, a semi-infinite needle can be viewed as the limit of an axially symmetric hyperboloidal tip with angle of aperture $\omega_{0}\to 0$. We point out that in the hyperboloidal problem, λ_1_ becomes arbitrarily small when $\omega_{0}\to 0$, both being well-defined and finite as long as *ω*_0_≠0. However, for *ω*_0_=0, the boundary condition on the tip cannot be fulfiled, as the equation $P_{\lambda_{1}}(\cos\pi )=0$ has no solution (in particular, λ_1_=0 is *not* a solution of this equation). This result shows that it is impossible to run a junction between a truly semi-infinite *one-dimensional* wire and a planar counterelectrode at a *finite* potential difference, at least in a three-dimensional space.

**(d) Paraboloid of revolution**

*Differential equations and general solution*. We now model the microtip as paraboloid of revolution by introducing three-dimensional parabolic coordinates [31] (figure 4)

A 41 $$\begin{array}{rl} & x=uv\cos\phi ,\\ & y=uv\sin\phi \\ \text{and}\; & z^{\prime }=\frac{u^{2}-v^{2}}{2}, \end{array}\}$$

where u,v∈[0,∞[ and *ϕ*∈[0,2*π*[. Surfaces of constant *u* are paraboloids of revolution with negative curvature, the limiting case *u*=0 corresponding to the negative *z*-axis. Surfaces of constant *v* are paraboloids of revolution with positive curvature, where the limiting case *v*=0 corresponds to the negative *z*-axis. The microtip is taken to be a paraboloidal surface of constant *u*=*u*_0_ or, equivalently, it is described by the equation *z*′=−(1/2)(*r*/*R*_0_)+*R*_0_/2, where $R_{0}≐u_{0}^{2}$ is the radius of curvature of the paraboloid. The coordinates of the tip apex are *x*=*y*=0 and *z*′_0_=*u*_0_/2. The Laplace equation in these coordinates reads

A 42 $$\frac{1}{u^{2}+v^{2}}\left\{\frac{1}{u}\frac{\partial }{\partial u}\left(u\frac{\partial }{\partial u}\right)+\frac{1}{v}\frac{\partial }{\partial v}\left(v\frac{\partial }{\partial v}\right)\right\}+\frac{1}{u^{2}v^{2}}\frac{\partial^{2}}{\partial \phi^{2}}\Phi =0.$$

By exploiting the rotationally symmetric Ansatz *Φ*(*u*,*v*,*ϕ*)=*U*(*u*)⋅*V* (*v*), we obtain the following differential equations:

A 43 $$\begin{array}{rl} & u^{2}U^{\prime \prime }(u)+uU^{\prime }(u)-n^{2}u^{2}U(u)=0\\ \text{and}\; & v^{2}V^{\prime \prime }(v)+vV^{\prime }(v)+n^{2}v^{2}V(v)=0, \end{array}\}$$

where *n*^2^ is the separation constant. The solution of equation (A 43) can be written in terms of Bessel functions *J*_0_(*nu*) and *Y*_0_(*nu*) and modified Bessel functions *I*_0_(*nv*) and *K*_0_(*nv*)) of order 0

A 44 $$\begin{array}{rl} & \Phi (u,v)=\sum_{k}[A_{n_{k}}J_{0}(n_{k}u)I_{0}(n_{k}v)+B_{m_{k}}Y_{0}(m_{k}u)I_{0}(m_{k}v)]\\ \text{and}\; & \Phi_{d}=\sum_{k}\left[A_{n_{k}}J_{0}\left(n_{k}\sqrt{2z_{P}^{\prime }+v^{2}}\right)I_{0}(n_{k}v)+B_{m_{k}}Y_{0}\left(m_{k}\sqrt{2z_{P}^{\prime }+v^{2}}\right)I_{0}(m_{k}v)\right], \end{array}\}$$

where *n*_*k*_ and *m*_*k*_ are defined by *J*_0_(*n*_*k*_*u*_0_)=0 and *Y*_0_(*m*_*k*_*u*_0_)=0, as a consequence of implementing the boundary condition *Φ*(*u*_0_,*v*)=0 on the tip surface. To obtain equation (A 44), we have implemented the boundary condition on the plane (*u*^2^−*v*^2^)/2=*z*′_P_ as well.

**(vi) Scaling behaviour: near planes** $\left(\sqrt{2z_{P}^{\prime }}-u_{0}\geq u_{0}\right)$.

We consider the boundary condition along the line *v*=0 and exploit the fact that for near planes, $\sqrt{2z_{P}^{\prime }}$ is close to *u*_0_. Because the functions *J*_0_(*n*_*k*_*u*) and *Y*_0_(*m*_*k*_*u*) vanish at *u*_0_, we have to lowest order

A 45 $$\begin{array}{rl} & J_{0}\left(n_{k}\sqrt{2z_{P}^{\prime }}\right)∼\left[\sqrt{2z_{P}^{\prime }}-u_{0}\right]\\ \text{and}\; & Y_{0}\left(m_{k}\sqrt{2z_{P}^{\prime }}\right)∼\left[\sqrt{2z_{P}^{\prime }}-u_{0}\right]. \end{array}\}$$

These equations imply the scaling behaviour

A 46 $$A_{n_{k}},B_{n_{k}}∼\Phi_{d}\frac{z_{0}^{\prime }}{z_{P}^{\prime }-z_{0}^{\prime }}$$

and the potential close to the tip apex and along the *z*′-axis reads, therefore,

A 47 $$\Phi (z^{\prime })∼\Phi_{d}\cdot \frac{\left(z^{\prime }-z_{0}^{\prime }\right)}{z_{P}^{\prime }-z_{0}^{\prime }}.$$

In terms of the *x*,*y* and *z*-coordinates, equation (A 47) recovers equation (2.5) exactly.

**(vii) Scaling behaviour: distant planes** $\left(\sqrt{2z_{P}^{\prime }}-u_{0}\gg u_{0}\right)$

The formulation of a scaling law in the case of a distant planar counterelectrode is difficult, because *J*_0_ and *Y*_0_ share the same asymptotic behaviour and are both oscillating, so that cancellations might occur. However, a strategy which foresees to approximate a large but finite distant plane with the flat portion of a confocal paraboloid $u=u_{1}=\sqrt{2z_{P}^{\prime }}$ might give a useful approximation. This specific choice of the counterelectrode geometry has the advantage that boundary conditions are homogeneous in the coordinate *u*, such that the solution solely depends on the variable *u*. Under these circumstances, equation (A 42) simplifies to

A 48 $$u^{2}\Phi^{\prime \prime }(u)+u\Phi^{\prime }(u)=0.$$

By accounting for the boundary conditions on the tip and on the parabolic counterelectrode, the solution of equation (A 48) reads

A 49 $$\Phi (u)=\frac{\Phi_{d}}{\ln(u_{1}/u_{0})}\cdot \ln\left(\frac{u}{u_{0}}\right).$$

Close to *u*_0_ and along the tip axis, the potential reads, therefore,

A 50 $$\Phi (z^{\prime })=\Phi_{d}\cdot \frac{1}{\ln(z_{P}^{\prime }/z_{0}^{\prime })}\cdot \frac{z^{\prime }-z_{0}^{\prime }}{z_{0}^{\prime }},$$

which is equivalent to equation (2.5) in terms of the coordinates *x*,*y* and *z*.

**(e) Cuspidal tip**

*Differential equations.* In this case, it is convenient to introduce bi-spherical coordinates (*α*,*β*,*ϕ*) (figure 2), defined through [32]

$$\begin{array}{rl} x & =\frac{a\sin\alpha \cos\phi }{\cosh\beta -\cos\alpha },\\ y & =\frac{a\sin\alpha \sin\phi }{\cosh\beta -\cos\alpha }\\ z^{\prime } & =\frac{a\sinh\beta }{\cosh\beta -\cos\alpha }, \end{array}$$

where 0≤*α*≤*π*, −∞<β<∞, 0≤*ϕ*≤2*π* and *a* is the focal distance. Surfaces of constant *α* are tori with radius a/sin⁡α and centre on the *z*′=0-plane at a distance acot⁡α from the origin of the coordinate system

A 51 $${\left(\sqrt{x^{2}+y^{2}}-a\cot\alpha \right)}^{2}+z^{\prime 2}=\frac{a^{2}}{\sin^{2}\alpha },$$

whereas surfaces of constant *β* are spheres of radius a/sinh⁡β centred at (0,0,acoth⁡β)

A 52 $${\left(z^{\prime }-a\coth\beta \right)}^{2}+(x^{2}+y^{2})=\frac{a^{2}}{\sinh^{2}\beta }.$$

In these coordinates, the tip surface is given by *α*=*α*_0_>0, such that the associated boundary condition is *Φ*(*α*_0_,*β*,*ϕ*)=0. The corresponding Laplace equation is separable if the following rotationally symmetric Ansatz is used

$$\Phi (\alpha ,\beta )=\sqrt{2\cosh\beta -2\cos\alpha }\cdot A(\alpha )\cdot B(\beta ).$$

The functions *A*(*α*) and *B*(*β*) satisfy the following equations:

A 53 $$\begin{array}{rl} & \frac{1}{\sin\alpha }\frac{d}{d\alpha }\left(\frac{1}{\sin\alpha }\frac{dA}{d\alpha }\right)+\lambda (\lambda +1)A=0\\ \text{and}\; & \frac{d^{2}B}{d\beta^{2}}={\left(\lambda +\frac{1}{2}\right)}^{2}B. \end{array}\}$$

with solution

A 54 $$\begin{array}{rl} \Phi (\alpha ,\beta ) & =\sqrt{2\cosh\beta -2\cos\alpha }\\ & \;\times \sum_{\lambda }[A_{\lambda }P_{\lambda }(-\cos\alpha )+B_{\lambda }Q_{\lambda }(\cos\alpha )]\\ & \;\times [C_{\lambda }\;e^{(\lambda +1/2)\beta }+D_{\lambda }\;e^{-(\lambda +1/2)\beta }]. \end{array}$$

Divergences at *α*=*π* (−*a*≤*z*′≤*a*) are avoided by setting *B*_λ_=0.

*Boundary condition on the tip.* By implementing the boundary condition on the tip, we obtain the equation $P_{\lambda }(-\cos\alpha_{0})=0$, with solutions λ_1_,λ_2_,…. These indices coincide with those computed for the conical tip model with aperture angle *ω*_0_=2*α*_0_.

*Scaling properties.* Let us consider a plane at *z*′=*z*′_P_ close to tip apex. Note that this plane cannot extend indefinitely in the *xy*-direction, because it would encounter the boundary represented by the tip itself, which is kept at a different potential. Relevant in this case is the behaviour of the solution for β→−∞. Under these circumstances, $\sinh(\beta )∼e^{-\beta }/2$ and coth⁡(β)∼−1, such that equation (A 52) simplifies to

A 55 $$(x^{2}+y^{2})+{\left(z_{P}^{\prime }+a\right)}^{2}=\frac{{\left(2a\right)}^{2}}{e^{-2\beta }},$$

which is the equation of a sphere with radius 2*a* *e*^*β*^ centred at the tip apex. Note that for the above approximation to hold, *z*′_P_ needs to lie in the interval [−*a*,0]. Along the tip axis, equation (A 55) implies $\beta \approx \ln((z_{P}^{\prime }+a)/2a)$, such that we must set *D*_λ_=0 in equation (A 54) to avoid divergence at $z_{P}^{\prime }\to -a$. The boundary condition along the axis reads accordingly

A 56 $$\Phi_{d}=\sum_{k=1}^{\infty }C_{\lambda_{k}}(\Phi_{d},z_{P}^{\prime })P_{\lambda_{k}}(1){\left(\frac{z_{P}^{\prime }+a}{a}\right)}^{\lambda_{k}},$$

wherefore

A 57 $$C_{\lambda_{k}}∼\Phi_{d}{\left(\frac{a}{z_{P}^{\prime }+a}\right)}^{\lambda_{k}}.$$

Finally, the leading order term of the potential along the tip axis and in the vicinity of the apex reads

A 58 $$\Phi (z^{\prime })∼\Phi_{d}{\left(\frac{a}{z_{P}^{\prime }+a}\right)}^{\lambda_{1}}{\left(\frac{z^{\prime }+a}{a}\right)}^{\lambda_{1}},$$

which is equivalent to equation (2.2) in terms of *x*,*y* and *z* (recall: *z*′_P_+*a*=*d*, figure 2).

## Appendix B. The tunnelling current density

In this appendix, we present the details regarding the computation of the integrals appearing in *G*(*E*_f_) and *J*_0_(*E*_f_).

**(a) Non-analytic potential**

The potential within the tunnelling barrier writes, to leading order, *Φ*(*z*)=*A*_λ_1__⋅*Φ*_*d*_⋅(*z*/*d*)^λ_1_^, where *A*_λ_1__ is a dimensionless constant. Referring to figure 5, we set *z*_1_=0 and

B 1 $$z_{2}=d{\left(\frac{\varphi }{A_{\lambda_{1}}e\Phi_{d}}\right)}^{1/\lambda_{1}}.$$

By introducing the dimensionless variable

B 2 $$y=\frac{z}{d}{\left(\frac{A_{\lambda_{1}}e\Phi_{d}}{\varphi }\right)}^{1/\lambda_{1}},$$

we obtain

B 3 $$\begin{array}{rl} G(E_{f}) & =2\sqrt{\frac{8\pi^{2}m}{h^{2}}}\int_{0}^{z_{2}}\sqrt{\left(\varphi -A_{\lambda_{1}}e\Phi_{d}{\left(\frac{z}{d}\right)}^{\lambda_{1}}\right)}dz\\ & =2\sqrt{\frac{8\pi^{2}m\varphi }{h^{2}}}\cdot d{\left(\frac{\varphi }{A_{\lambda_{1}}e\Phi_{d}}\right)}^{1/\lambda_{1}}\int_{0}^{1}\left(\sqrt{1-y^{\lambda_{1}}}\right)dy\\ & =2\sqrt{\frac{8\pi^{2}m\varphi }{h^{2}}}\cdot d{\left(\frac{\varphi }{A_{\lambda_{1}}e\Phi_{d}}\right)}^{1/\lambda_{1}}\cdot \frac{\sqrt{\pi }\cdot \Gamma (1+1/\lambda_{1})}{2\cdot \Gamma (3/2+1/\lambda_{1})}\\ & =2\pi^{3/2}\frac{\Gamma (1+1/\lambda_{1})}{\Gamma (3/2+1/\lambda_{1})}\cdot {\left(\frac{A_{\lambda_{1}}e\Phi_{d}}{\varphi }\cdot {\left(\frac{d}{\Lambda_{\varphi }}\right)}^{-\lambda_{1}}\right)}^{-1/\lambda_{1}} \end{array}$$

and, similarly,

B 4 $$\begin{array}{rl} J_{0}(E_{f}) & =\frac{4\pi em}{h^{3}}{\left(\sqrt{\frac{8\pi^{2}m}{h^{2}}}\frac{d\cdot \varphi^{1/\lambda_{1}-1/2}}{{\left(A_{\lambda_{1}}e\Phi_{d}\right)}^{1/\lambda_{1}}}\cdot \int_{0}^{1}\frac{1}{\sqrt{1-y^{\lambda_{1}}}}\;dy\right)}^{-2}\\ & =\frac{4\pi em}{h^{3}}{\left(\sqrt{\frac{8\pi^{2}m}{h^{2}}}\frac{d\cdot \varphi^{1/\lambda_{1}-1/2}}{{\left(A_{\lambda_{1}}e\Phi_{d}\right)}^{1/\lambda_{1}}}\cdot \frac{\sqrt{\pi }\Gamma (1+1/\lambda_{1})}{\Gamma (1/2+1/\lambda_{1})}\right)}^{-2}\\ & =\frac{e\cdot \varphi }{h\cdot \Lambda_{\varphi }^{2}}\cdot {\left(\frac{\Gamma (1/2+1/\lambda_{1})}{\sqrt{2}\pi \Gamma (1+1/\lambda_{1})}\right)}^{2}\cdot {\left(\frac{A_{\lambda_{1}}e\Phi_{d}}{\varphi }\cdot {\left(\frac{d}{\Lambda_{\varphi }}\right)}^{-\lambda_{1}}\right)}^{2/\lambda_{1}}. \end{array}$$

We observe that both the expressions for *G*(*E*_f_) and *J*_0_(*E*_f_) depend on the scaling variable *Φ*_*d*_ *d*^−λ_1_^, whose natural unit turns out to be $\varphi \;\Lambda_{\varphi }^{-\lambda_{1}}/e$.

**(b) Rounded tips: linear potential**

In this case, the potential is given by *Φ*(*z*)=*A*_λ_1__(*a*/*d*)^λ_1_^⋅*Φ*_*d*_⋅*z*/*a* for distant planes providing the boundary condition and by *Φ*(*z*)=*A*_1_⋅*Φ*_*d*_⋅*z*/*d* for near planes. Therefore, we set *z*_1_=0 and

B 5 $$\begin{array}{rl} & z_{2}=\frac{d\cdot \varphi }{A_{\lambda_{1}}e\Phi_{d}}\;\text{(near plane)}\\ \text{and}\; & z_{2}=\frac{a\cdot \varphi }{A_{\lambda_{1}}e\Phi_{d}{\left(a/d\right)}^{\lambda_{1}}}\;\text{(distant plane)}. \end{array}\}$$

The computation of *G*(*E*_f_) and *J*_0_(*E*_f_) reduces to the computation of the elementary integrals

B 6 $$\int_{0}^{1}\left(\sqrt{1-y^{1}}\right)dy\;\mathrm{and}\;\int_{0}^{1}\frac{1}{\sqrt{1-y^{1}}}\;dy,$$

such that one obtains

B 7 $$\begin{array}{rl} & G^{\mathrm{near}}(\Phi_{d},d)=\frac{8\pi }{3}\cdot \frac{\varphi }{A_{1}e\Phi_{d}\cdot \Lambda_{\varphi }/d},\\ & J_{0}^{\mathrm{near}}(\Phi_{d},d)=\frac{1}{8\pi }\cdot \frac{e\cdot \varphi }{h\cdot \Lambda_{\varphi }^{2}}\cdot \frac{{\left(A_{1}e\Phi_{d}\cdot \Lambda_{\varphi }/d\right)}^{2}}{\varphi^{2}},\\ & G^{\mathrm{distant}}(\Phi_{d},d)=\frac{8\pi }{3}\cdot \frac{a}{\Lambda_{\varphi }}\cdot \frac{\varphi }{A_{\lambda_{1}}e\Phi_{d}\cdot {\left(a/d\right)}^{\lambda_{1}}}\\ \text{and}\; & J_{0}^{\mathrm{distant}}(\Phi_{d},d)=\frac{1}{8\pi }\cdot \frac{e\cdot \varphi }{h\cdot \Lambda_{\varphi }^{2}}\cdot \frac{\Lambda_{\varphi }^{2}}{a^{2}}\cdot \frac{{\left(A_{\lambda_{1}}e\Phi_{d}\cdot {\left(a/d\right)}^{\lambda_{1}}\right)}^{2}}{\varphi^{2}}. \end{array}\}$$

**(c) The appearance of *Λ*_*φ*_**

A most remarkable feature common to all equations in this appendix is the appearance of a characteristic length *Λ*_*φ*_, which is the De Broglie wavelength associated with an electron with an energy corresponding to the potential energy barrier *φ*. The simplest way to illustrate this somewhat surprising appearance of *Λ*_*φ*_ is to consider the Gamov factor for the elementary triangular barrier in the planar geometry [33]

B 8 $$G^{\mathrm{el}}=\frac{8\pi }{3}\cdot \frac{\sqrt{2m}}{e\cdot h}\frac{\varphi^{3/2}}{F},$$

where *F* is the electric field and the superscript el used in ref. [33] probably stands for ‘elementary’. In the planar geometry, one has *F*=*Φ*_*d*_/*d*. Accordingly, *G*^*el*^ writes

B 9 $$G^{\mathrm{el}}=\frac{8\pi }{3}\cdot \frac{\sqrt{2m}}{h}\cdot d\cdot \frac{\varphi^{3/2}}{e\Phi_{d}}.$$

Note that the Gamov factor must be dimensionless, as it is an exponent. To rewrite *G*^*el*^ as a manifestly dimensionless quantity proceed as follows:

(i) *φ*^3/2^ is written as *φ*⋅*φ*^1/2^.
(ii) The first factor *φ* has the units of energy and is used to cancel the dimension of *eΦ*_*d*_.
(iii) The second factor *φ*^1/2^ is associated with the remaining terms to create a quantity with the dimensions of an inverse length, which cancels the dimension of *d*. One finds that this quantity is exactly the inverse of the characteristic length *Λ*_*φ*_.

This shows that *Λ*_*φ*_ was just hidden in the widely known expression for *G*^*el*^. Having (proudly) demonstrated the appearance of *Λ*_*φ*_ in the Gamov exponent, we discovered that the same length was already introduced into a Gamov exponent more than 80 years ago by J. R. Oppenheimer (eqn (1) in ref. [12])!
